# Application of Multiple Regression and Design of Experiments for Modelling the Effect of Monoethylene Glycol in the Calcium Carbonate Scaling Process

**DOI:** 10.3390/molecules23040860

**Published:** 2018-04-10

**Authors:** Vinicius Kartnaller, Fabrício Venâncio, Francisca F. do Rosário, João Cajaiba

**Affiliations:** 1 Instituto de Química, Pólo de Xistoquímica, Universidade Federal do Rio de Janeiro (UFRJ), Rua Hélio de Almeida 40, Cidade Universitária, Rio de Janeiro 21941-614, Brazil; vkartnaller@yahoo.com.br (V.K.); fabricioqv@yahoo.com.br (F.V.); 2 Centro de Pesquisas e Desenvolvimento Leopoldo Américo Miguez de Mello, PETROBRAS, Cidade Universitária, Rio de Janeiro 21040-000, Brazil; frosario@petrobras.com.br

**Keywords:** calcium carbonate, gas hydrate, scale, monoethylene glycol, MEG, flow assurance

## Abstract

To avoid gas hydrate formation during oil and gas production, companies usually employ thermodynamic inhibitors consisting of hydroxyl compounds, such as monoethylene glycol (MEG). However, these inhibitors may cause other types of fouling during production such as inorganic salt deposits (scale). Calcium carbonate is one of the main scaling salts and is a great concern, especially for the new pre-salt wells being explored in Brazil. Hence, it is important to understand how using inhibitors to control gas hydrate formation may be interacting with the scale formation process. Multiple regression and design of experiments were used to mathematically model the calcium carbonate scaling process and its evolution in the presence of MEG. It was seen that MEG, although inducing the precipitation by increasing the supersaturation ratio, actually works as a scale inhibitor for calcium carbonate in concentrations over 40%. This effect was not due to changes in the viscosity, as suggested in the literature, but possibly to the binding of MEG to the CaCO_3_ particles’ surface. The interaction of the MEG inhibition effect with the system’s variables was also assessed, when temperature’ and calcium concentration were more relevant.

## 1. Introduction

In a reservoir, oil can be mixed with water, salts and gas, and all phases are in equilibrium under high temperature and pressure conditions. During production, these fluids undergo differences in the system’s conditions that may lead to different problems related to fouling. Pressure drops and variations in temperature change the phase equilibrium of the fluids, enabling precipitation and accumulation of organic and/or inorganic solids. This fouling leads to loss of production or even to well loss.

Inorganic fouling, also known as scale, represents a big challenge during oil production, with an estimated US$1.4 billion spent for prevention and remediation in 2002 [1]. The most common inorganic salt responsible for scaling is calcium carbonate, especially in ultra-deep carbonate pre-salt fields [2]. This scale formation is highly pH-dependent and is caused by depressurization during production. Depressurization decreases the solubility of CO_2_ in water, which is in the form of carbonic acid and bicarbonate. Through the loss of the gas from the water phase, pH increases, and the calcium carbonate saturation may be achieved. Equations (1)–(3) show the CO_2_ equilibrium in water and the calcium carbonate precipitation equilibrium:

CO_2_(g) ⇆ CO_2_(aq) (1)

CO_2_(aq) + H_2_O ⇆ H_2_CO_3_(aq) ⇆ H^+^(aq) + HCO_3_^−^(aq) (2)

Ca^2+^(aq) + CO_3_^2−^(aq) ⇆ CaCO_3_(s) (3)

The tendency of this precipitation to occur depends on the ion concentrations and the equilibrium constant. The spontaneity of the precipitation can be measured by the supersaturation ratio (*SR*), presented in Equation (4):

(4) $$SR=a_{Ca^{2+}}\times a_{HCO_{3}^{-}}/K_{sp}$$

If *SR* < 1, the system is considered non-saturated and no precipitation occurs, while if *SR* ≥ 1, the system achieved saturation and precipitation is spontaneous.

Another type of fouling that is of great concern for off-shore oil production is gas hydrate formation [3,4]. Gas hydrates are crystalline solids consisting of gas molecules (usually methane and CO_2_) surrounded by water molecules [5]. This fouling is formed under high pressure and low temperature, like the conditions at sea-floor levels. Hydrates also have a financial impact. In 2002, it was estimated it would cost US$100 million to remediate or prevent hydrates formation [6]. However, remediation is not the best scenario for any types of fouling because it is more expensive. Prevention is the best option for production companies. Inhibitor molecules are used to affect the thermodynamic or the kinetics of the solids formation or to change the structure and characteristics of the solids formed. This ensures that deposits are not formed in the production line, guaranteeing flow assurance.

For gas hydrate fouling, inhibitors can specifically affect the thermodynamics or the kinetics of the crystallization process. Thermodynamic inhibitors are used in high quantities and consist of hydroxyl compounds that interact with water so that fewer molecules are free to form the hydrate. The main inhibitors of this type are monoethylene glycol (MEG), methanol and ethanol [7]. However, although they are effective for preventing gas hydrate formation, the fact that their molecules interact with water may affect scale formation as well. Since fewer water molecules are free to interact with the ions in the phase, the activity of the ions increases with the addition of the hydrate inhibitor. This increases the supersaturation ratio (Equation (4)), leading to more scale formation [8,9,10].

Past studies have shown that even though MEG increased the supersaturation ratio, it had a tendency to increase the induction time (time in which crystals are observed out of solution) and to decrease the kinetic growth of calcium carbonate [11,12,13,14,15,16]. This goes against the thermodynamic conclusion that the supersaturation ratio is the only defining response to predict scale formation, since even though MEG increased the tendency for precipitation, it also acted as an inhibitor of crystal growth. However, all the results regarding this effect were conducted in batch seeded or unseeded systems. The extension of this effect in scale formation is yet to be resolved, since the process is not only dependent on the crystallization, but also on the capacity of deposition and agglomeration in a flow line.

This work aims to study the scaling process of calcium carbonate in the presence of MEG to gain a better understanding of how this hydrate inhibitor may influence scale formation. For that, a design of experiment was used to acquire the maximum amount of information, and a modelling was made of the data measured over time. Since the response is time-dependent and all information are available at the end of the experiment, a multiple regression was used to evaluate the scaling process in different extensions. This approach enables a better of use of the data, extracting more information of processes that change with time [17].

## 2. Materials and Methods

Calcium chloride dihydrate was used to prepare a calcium stock solution. Dilutions were made from the resulting stock solution, followed by the addition of MEG. Sodium bicarbonate solutions were prepared daily, followed by the addition of MEG. All solutions were prepared using newly degassed and distilled water to ensure the solution would not absorb gaseous CO_2_ from the air.

The conditions in the tests followed a 2^5–1^ central composite rotational design (resolution V). Table 1 shows the experimental matrix, in which the independent variables under review (factors) were pressure, temperature, MEG amount, carbonate ion concentration and calcium ion concentration. To estimate the experimental variance, the central point was replicated six times (Experiments 17–22).

**Figure 1:**
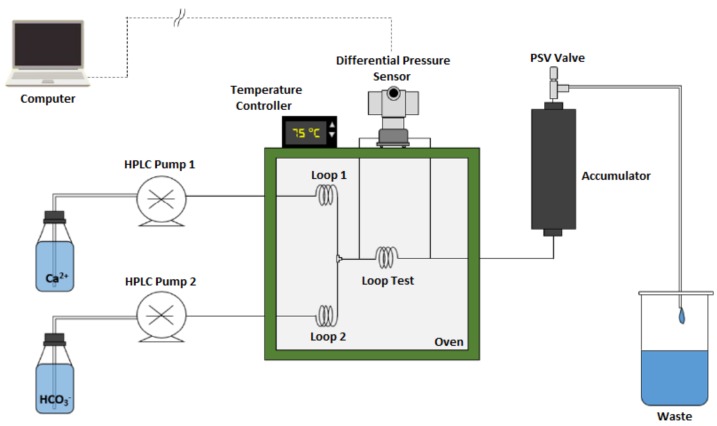
Scheme of the Dynamic Scale Loop (DSL) system used in the experiments.

Experiments were performed in a Dynamic Scale Loop (DSL) system. Figure 1 shows a scheme of the equipment. In the setup, two HPLC pumps pushed the newly prepared calcium chloride and sodium bicarbonate solutions into a thermostat-regulated oven through a 1.8 m stainless steel tube with an inner diameter of 1 mm. Loops A and B ensured that the solutions reached the mixture chamber at the correct temperature for the tests. After the solutions were mixed, the combination went through the loop test, which had the same dimensions of the previous tubes. The supersaturated solution was then achieved, which led to calcium carbonate formation and later deposition onto the tube’s wall. When deposition occurred, the inlet pressure became greater than the outlet pressure, which generated a differential pressure. The more deposit was formed, the higher the differential pressure and the more advanced the scaling process became. The injection flow rate was 10.0 mL min^−1^, in which 5.00 mL min^−1^ was set for each solution. Pressure of the system was regulated using a PSV valve connected outside the oven. The differential pressure was measured using a model EJA 130A high-static differential pressure transmitter (Yokogawa, Musashino, Tokyo, Japan).

The differential pressure data were acquired over time using a LabView-based software program and were used as the response for modelling the system. Since the differential pressure, related to the advancement of the scaling process, was measured over time, these data were used for multiple regression models. Each model was constructed using the time it took for the scaling to increase the differential pressure baseline in increments of 1 psi, going from 1 to 25 psi, for all the experiments. The subsequent data processing was performed using Matlab R2015b (MathWorks, Natick, MA, USA).

After the models were constructed, additional experiments were performed, in order to test its prediction power. The new experiments were made with different MEG concentrations, whilst the other factors were kept constant, in the conditions of the central point experiments (Experiments #17–22 in Table 1). The MEG concentrations used for the extra experiments were: 10%, 20%, 30%, 50%, 60% and 70%. It should be noted that the experiments containing 0%, 40% and 80% of MEG were already present in the central composite design (Experiments 27, 17–22 and 28, respectively).

## 3. Results

### 3.1. Data Acquision and Modelling

**Figure 2:**
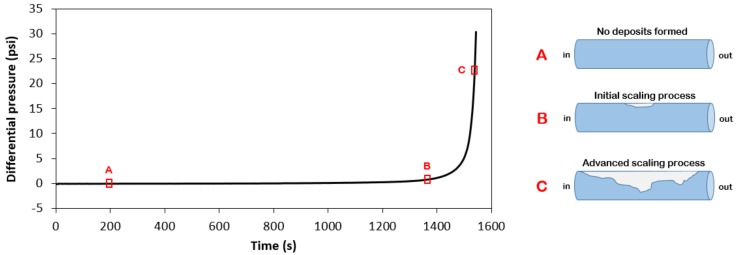
Graph of the measured differential pressure over time for experiment #1 (Table 1) and schematic representation of the advancement of the scale process in a line over different points in time.

The study of the calcium carbonate scaling process was achieved by evaluating a signal related to solid accumulation in the flow line. Although precipitation is mandatory in the process, it is not the only step for scale formation. The solids formed need to adhere to the tube’s wall, forming deposits. Once solids are deposited, they may start to grow and agglomerate, leading to advancement of the scaling. The differential pressure measures this development, since the accumulated solids increase the pressure in the inlet of the line. The two pumps are regulated to work at constant flow; hence, the scale deposit decreases the cross-sectional area of the loop test, causing a pressure increase. Figure 2 shows an example of the data measured over time for an experiment and illustrates the scaling formation.

Different system conditions may influence the beginning of scale formation and its development. Temperature, for an example, decreases calcium carbonate solubility and tends to decrease the time in which the scale is formed. This time, also called scaling time, depends on different variables such as temperature, ionic concentration, ionic strength, pressure, and pH. Finding an exact physical/chemical model using these different variables simultaneously is very difficult, since multiple correlations may exist between them and no exact equation has been proposed for that. Hence, for these multi-variable systems, the best way to describe it is empirically. For empirical models, the use of design of experiments is the best option since it can extract as much information as possible with the least number of experiments.

**Figure 3:**
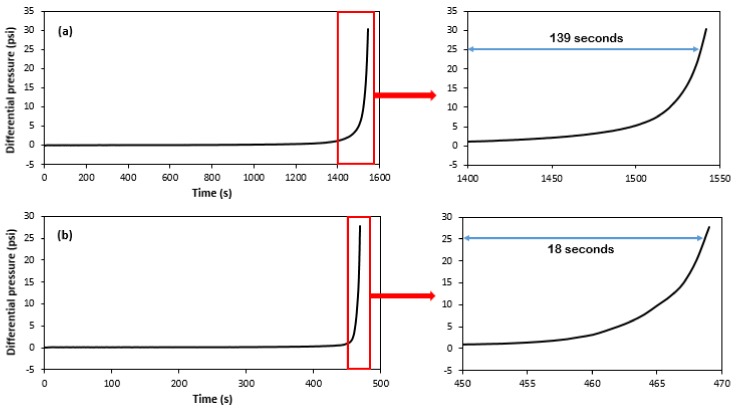
The measured differential pressure over time for (**a**) experiment #1 and (**b**) experiment #11 (Table 1), showing details about the speed evolution of the scale build-up process.

For this work, a central composite design was used to understand mainly how adding MEG (a gas hydrate inhibitor) may influence the calcium carbonate scale process. The independent variables, i.e., the variables that causes variation to the response, were the MEG concentration, calcium concentration, bicarbonate concentration, temperature and pressure. Equation (5) was used for modelling the system showing the “direct” and the quadratic dependency on these variables:

(5) $$\begin{array}{cc} \ln(t_{sc})=b_{0} & +(b_{1}\times P)+(b_{2}\times T)+(b_{3}\times MEG)+(b_{4}\times C_{HC{O_{3}}^{-}})+(b_{5}\times C_{Ca^{2+}})\\ & +(b_{12}\times P\times T)+(b_{13}\times P\times MEG)+(b_{14}\times P\times C_{HC{O_{3}}^{-}})\\ & +(b_{15}\times P\times C_{Ca^{2+}})+(b_{23}\times T\times MEG)+(b_{24}\times T\times C_{HC{O_{3}}^{-}})\\ & +(b_{25}\times P\times C_{Ca^{2+}})+(b_{34}\times MEG\times C_{HC{O_{3}}^{-}})\\ & +(b_{35}\times MEG\times C_{Ca^{2+}})+(b_{45}\times C_{HC{O_{3}}^{-}}\times C_{Ca^{2+}})+\left(b_{11}\times P\times P\right)\\ & +\left(b_{22}\times T\times T\right)+\left(b_{33}\times MEG\times MEG\right)+\left(b_{44}\times C_{HC{O_{3}}^{-}}\times C_{HC{O_{3}}^{-}}\right)\\ & +\left(b_{55}\times C_{Ca^{2+}}\times C_{Ca^{2+}}\right) \end{array}$$

where *P* is the pressure, *T* is the temperature, *MEG* is the *MEG* content, $C_{HC{O_{3}}^{-}}$ is the bicarbonate concentration, and $C_{Ca^{2+}}$ is the calcium concentration. The set ($b_{0}$, $b_{1}$, $b_{2},\ldots ,b_{44},b_{55}$) is the coefficients of the equation to be estimated using multiple linear regression. All the independent variables were coded, meaning they were normalized so they were at the same scale. The dependent variable, i.e., the response, $\ln(t_{sc})$, is the natural logarithm of the time for scale to be formed. The logarithm was used, instead of just the scaling time, since there is a big non-linear exponential relationship for the response, as can be seen in the Supplementary Materials. However, as discussed, scale formation is a process that varies over time, and defining it in just one point is throwing away a great deal of information. Modelling just the beginning or the end does not give an idea of the evolution of the process under different conditions. Figure 3 shows an example of this idea for two experiments of the design, in which the details regarding the difference in the evolution of the scale process as it plugs the line can be seen.

For different conditions, not only does the process begin at different times (it took almost 1400 s for experiment #1 to have a ΔP of 1 psi, while for the experiment #11, the time required for a ΔP of 1 psi was almost three times less), but how it builds up in the line is also affected. For the purposes of this work, it was considered a final advanced scale when the system reached a ΔP of 25 psi. Thus, it took 139 s for experiment #1 to go from the initial scale formation to an advanced formation, while it took only 18 s for experiment #11 to achieve the same build-up.

To best understand the scaling process, all the information should be explored and as much data should be used for the modelling as possible. Hence, the dependent response was chosen as the different time it took all experiments to reach different levels of scaling, going from the baseline to 1 psi, to 2 psi, and so on until 25 psi. This way, the dependent response was not a vector, but a matrix containing information of different points of the scaling process in its columns, leading to a multiple regression for 25 different dependent variables. Equation (6) shows the data matrix as a 32 by 25 matrix in which each column represents the scaling time for different levels of the process and each row the different experiments of the central composite design:

(6) $$Y=[\begin{array}{cccc} t_{\begin{array}{c} inc,1 \end{array}}^{\Delta P=1psi} & t_{\begin{array}{c} inc,1 \end{array}}^{\Delta P=2psi} & \cdots & t_{\begin{array}{c} inc,1 \end{array}}^{\Delta P=25psi}\\ t_{\begin{array}{c} inc,2 \end{array}}^{\Delta P=1psi} & t_{\begin{array}{c} inc,2 \end{array}}^{\Delta P=2psi} & \cdots & t_{\begin{array}{c} inc,2 \end{array}}^{\Delta P=25psi}\\ \begin{array}{c} \vdots \end{array} & \vdots & \cdots & \begin{array}{c} \vdots \end{array}\\ t_{inc,32}^{\Delta P=1psi} & t_{inc,32}^{\Delta P=2psi} & \cdots & t_{inc,32}^{\Delta P=25psi} \end{array}]$$

Using this data matrix, the linear regression was performed, and the coefficients from Equation (5) were calculated for all the 25 models. However, some coefficients ($b_{1}$, $b_{12}$, $b_{13}$, $b_{14}$, $b_{24}$, and $b_{35}$) were not significant and were eliminated from the model. The results of the regression and its statistical evaluation are presented as Supplementary Materials. It should be noted that all models showed a significant regression, with R^2^ and adjusted R^2^ close to 1 and with good statistics. These models were then used to estimate the scaling process and evaluate MEG’s influence on its own and its interactions with the other variables.

### 3.2. Evaluation of the MEG Effect over the Scaling Process

**Figure 4:**
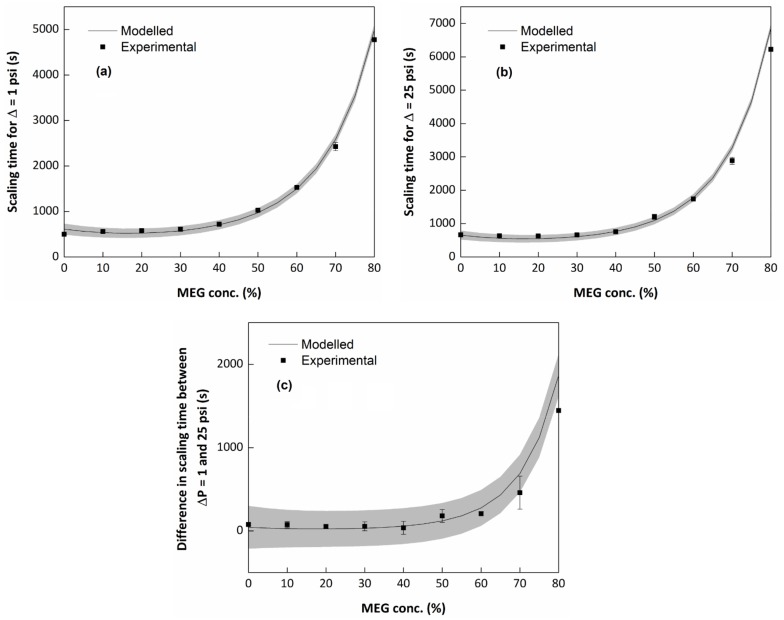
Modelled and experimental scaling time for different MEG concentrations in solution, where (**a**) scaling time to achieve ΔP = 1 psi (initial scale formation); (**b**) scaling time to achieve ΔP = 25 psi (advanced scale formation); and (**c**) difference between the scaling time to achieve ΔP = 1 psi and ΔP = 25 psi.

As described in the literature, MEG has an effect on calcium carbonate’s crystal growth and agglomeration for batch crystallization system. This behaviour was also perceived in the experiments performed in a flow system. Results from the modelling showed that a high amount of MEG added to the solutions led to an overall increase in the scaling time. To check these results, additional experiments were performed in triplicates with varying MEG concentration, whilst the other variables were kept constant at the central point conditions of the design of experiments (Table 1). The experimental and modelled results are shown in Figure 4. It should be noted that three of the experimental points were already present in the modelling, since they were part of the central and axial points of the design of experiments: 0% MEG (experiment #27), 40% MEG (experiments #17–22) and 80% MEG (experiments #28).

Both the initial scale formation (for a variation in the baseline of ΔP = 1 psi) and the advanced scale formation (for a variation in the baseline of ΔP = 25 psi) suffered a big effect by the MEG addition. The magnitude of the scaling time varied from five to six times compared to the blank experiment when the amount of MEG added was 80%. However, for lower MEG concentrations (up to a concentration of 40%), the modelled scaling time appeared not to vary much or to decrease a small amount. This could be an effect of the changes in the supersaturation ratio by the MEG addition. It appears that there are two effects controlling the system: one ruled by the supersaturation ratio and another ruled by an inhibition phenomenon. For the system with a concentration lower than 40%, the dispute between the two effects leads to small changes in the scaling time; however, for systems with a concentration of more than 40%, the inhibition effect tends to be bigger than the effect saturation effect, increasing the scaling time with the increase of the MEG concentration.

**Figure 5:**
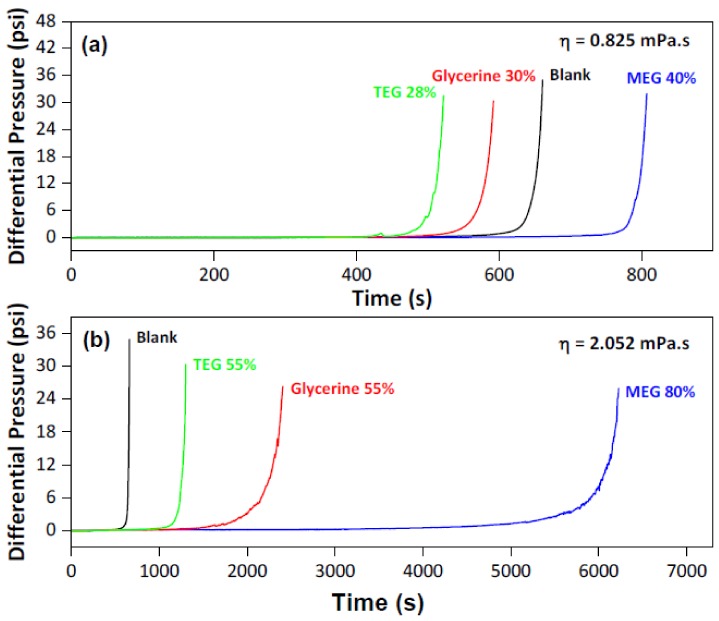
Differential pressure profiles for comparison of different systems with the same viscosity, where the system’s viscosities are: (**a**) 0.825 mPa s, and (**b**) 2.052 mPa s.

For concentrations over 40%, the addition of MEG not only affected the initial formation, but also the evolution of the process. Figure 4c shows the time difference between the initial scale formation (Figure 4a) and the advanced scale formation (Figure 4b). The MEG added influenced the time in which it took the system to go from the beginning of the scaling to the end. This means that MEG makes it harder for the solids to deposit and agglomerate in the line, which is correlated to the results achieved in the batch crystallization experiments. In these experiments, the effect of MEG in the calcium carbonate growth is often associated with changes in the medium’s viscosity [11,15]. To evaluate that, additional experiments were carried out using two other compounds with high viscosity (triethylene glycol and glycerin), in conditions in which the system’s viscosity was the same as in the experiments of the central and axial points of the design of experiment. Figure 5 shows the results.

For the systems with the lower viscosity, comparable to the experiment with 40% MEG, both triethylene glycol (TEG) and glycerin have shown a lower scaling time. The process using these two components developed even faster than the blank experiment (without any hydrate inhibitor), which might show that supersaturation was controlling the system. For the experiments with higher viscosity, comparable to the experiment with 80% MEG, TEG and glycerin showed a scaling time higher than the blank, but still lower than with MEG. However, in this case, these compounds acted as scale formation inhibitors. There seems to have been a minimum concentration of these inhibitor molecules in which the supersaturation stops controlling the system and inhibition occurs. Since viscosity was the same for the three different systems and yet the scaling time was different, it is not the main effect over its deposition and agglomeration.

An explanation of this overall behaviour can come from biomineralization, which is the production of hard inorganic parts by organisms that is still a scientific mystery in many aspects. One area still to understand in this subject is how organisms can affect and control mineral growth. For example, calcites (calcium carbonate’s most stable polymorph) grown in pure solution present a dramatic crystalline difference from those grown by mineralization [18]. This crystal growth control is usually attributed to complex organic molecules, known as coccolith-associated polysaccharides (CAPs) [19]. These are large polymeric carbohydrate molecules containing a variety of functional groups, such as OH and COOH. Many studies have tried to model these interactions, probing that indeed molecules containing these groups, such as alcohols, can bind to specific calcite crystal faces, which may lead to control of crystal growth [20,21,22,23]. Since the molecules studied in the present work are also hydroxyl compounds, there may be an interaction with the particles’ surface, controlling the crystal growth, which would explain the results. However, this can so far only be suggested. Further studies should be performed to best understand the MEG effect, which goes beyond this work.

### 3.3. Evaluation the System’s Variables over the MEG Effect

**Figure 6:**
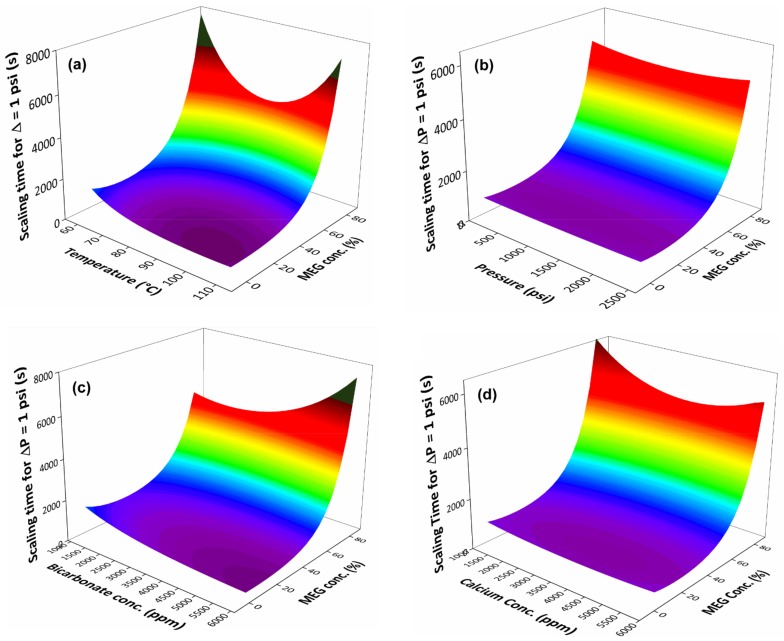
Modelled response surface of the initial scaling time for varying MEG concentration and: (**a**) temperature, (**b**) pressure, (**c**) bicarbonate concentration, and (**d**) calcium concentration.

It is also important to assess how the system’s variables can interact with the MEG effect and possibly decrease its inhibition power. The studied variables in this work were temperature, pressure, calcium concentration and bicarbonate concentration. Using the models developed by the linear regression, the scaling time was calculated for varying concentrations of MEG and one of the system’s variables, one at a time, while the other variables were kept constant at the central point values (from experiments #17–22 in Table 1). Figure 6 shows the response surface for varying MEG concentration and the system’s variables, for the initial scaling time.

**Figure 7:**
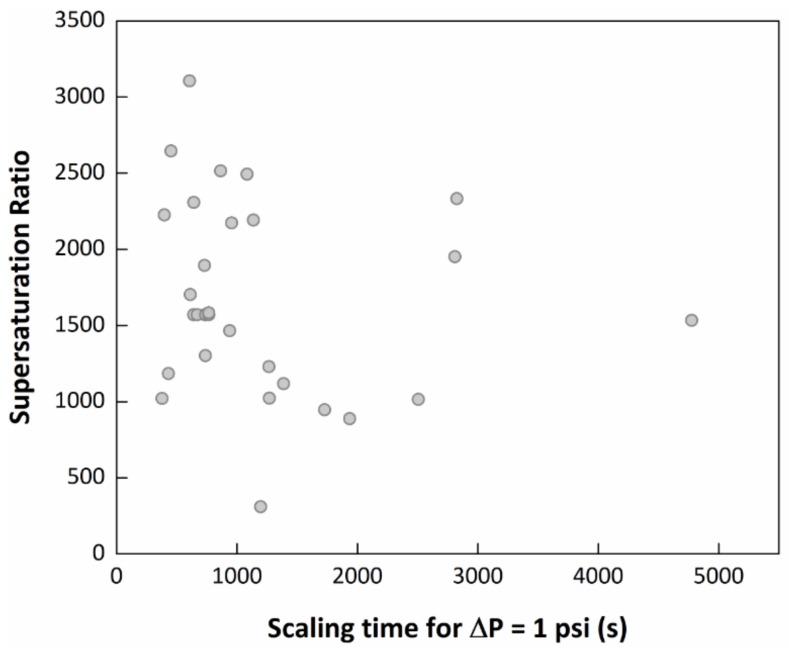
Graph of the scaling time versus the supersaturation ratio for the experiments in the design of experiment.

From the response surface results, it was seen in Figure 6b that pressure did not show much interaction with the MEG inhibition effect and over the scaling time. Temperature, however, had the most significant interaction. This may be due to temperature having a bigger individual effect over the entire scaling process, since higher temperature leads to a smaller calcium carbonate solubility and faster scale formation. This behaviour is seen in Figure 6a, for a part of the surface, in which the increase in temperature leads to a faster scaling. However, for higher amounts of MEG added to the solution, this behaviour seemed to change. The modelling results showed that for a system containing 80% of MEG, an increase in temperature up to around 100 °C decreased the scaling time, as expected; however, for higher temperatures, scaling time appeared to increase again, showing a synergy with the MEG inhibition effect, which might also be related to calcium carbonate solubility in this binary system. Similar effect was seen for the interaction between the bicarbonate and calcium concentration with the MEG concentration, in Figure 6c,d. At low MEG content, the increase of the ion concentration led to a decrease in the scaling time, due to the increase of the supersaturation ratio, as expected. However, for high MEG content, this behaviour changes for both graphs, in which for higher ions concentration, the scaling time increases, also showing a synergy with the MEG inhibition effect. This result is not expected, since it shows that there is no correlation between the supersaturation ratio and the scaling time. In fact, by plotting the initial scaling time (ΔP = 1 psi), calculated by the commercial software MultiScale™, against the supersaturation ratio for the experiments of the design of experiment, no dependence is seen. This suggests that the scale formation is not directly dependent on supersaturation for this type of system, which can be seen in Figure 7.

This behaviour cannot be directly understood and the interaction between the ion concentrations and the MEG concentration may be correlated to the solvation of the ions in solution, in which the dynamics change as the binary-solvents change. More studies should be made to try to deepen the understanding of the species in solution and how it affects nucleation and growth in the glycol-water system.

## 4. Conclusions

A better understanding on the role of monoethylene glycol in calcium carbonate scale formation was given. Multiple regression and design of experiments were used to mathematically model the scaling process in a dynamic pressurized system. MEG showed an inhibition effect for concentrations over 40% by affecting crystal growth and agglomeration in the flow line. This reflected in an increase of the initial scaling time and also in how fast the scaling process advanced. This effect is suggested in the literature as being due to changes in the system’s viscosity. However, it was shown that viscosity does not play as the only factor in this phenomenon. A suggestion was made that the MEG molecules were somehow bonding to the calcium carbonate particles’ surface being formed in the system, creating a hydrophobic layer and changing its capacity to interact among themselves and agglomerate. This behaviour is observed in the biomineralization systems, in which organisms form calcium carbonate with controlled growth due to the presence of molecules containing OH bonds. The effect interaction of MEG and other system variables were also assessed, and pressure did not show much influence over the MEG inhibition effect. Temperature, however, showed a big interaction with this MEG effect, probably due to its big influence in the system by itself, in which a synergy with the MEG inhibition effect was seen. Bicarbonate and calcium concentration also showed an interaction with the MEG effect, in which in systems containing high amounts of MEG, an increase in their concentration led to a slower scale formation, contrary to expectations. This was suggested as part of a different ionic solvation in the binary-solvent system, and further study should be conducted to understand this interaction.

## Figures and Tables

**Table 1:** Experimental table of the central composite design.

Experiment #	Pressure (psi)	Temperature (°C)	MEG Conc. (*v*/*v* %)	$C_{HC{O_{3}}^{-}}$ (ppm)	$C_{Ca^{2+}}$ (ppm)
1	714	60	23	2449	4551
2	1751	60	23	2449	2449
3	714	90	23	2449	2449
4	1751	90	23	2449	4551
5	714	60	57	2449	2449
6	1751	60	57	2449	4551
7	714	90	57	2449	4551
8	1751	90	57	2449	2449
9	714	60	23	4551	2449
10	1751	60	23	4551	4551
11	714	90	23	4551	4551
12	1751	90	23	4551	2449
13	714	60	57	4551	4551
14	1751	60	57	4551	2449
15	714	90	57	4551	2449
16	1751	90	57	4551	4551
17–22	1233	75	40	3500	3500
23	0	75	40	3500	3500
24	2466	75	40	3500	3500
25	1233	40	40	3500	3500
26	1233	110	40	3500	3500
27	1233	75	0	3500	3500
28	1233	75	80	3500	3500
29	1233	75	40	1000	3500
30	1233	75	40	6000	3500
31	1233	75	40	3500	1000
32	1233	75	40	3500	6000

## Supplementary Materials

The experimental design matrix, the response matrix, the statistical results for the modelling and the polymorphic evaluation of the calcium carbonate crystals are available online.
