# Supplementary material for: Application of Multiple Regression and Design of Experiments for Modelling the Effect of Monoethylene Glycol in the Calcium Carbonate Scaling Process
Source: Molecules. 2018 Apr 10;23(4):860. doi: 10.3390/molecules23040860 (PMC6017891; doi:10.3390/molecules23040860)
Supplement: Supplementary file 1 [file molecules-23-00860-s001.pdf]

SUPPLEMENTARY INFORMATION

Application of Multivariate Multiple Regression and Design of Experiment for Modelling the Effect of Monoethylene Glycol in the Calcium Carbonate Scaling Process

Vinicius Kartnaller[1], Fabrício Venâncio[1], Francisca F. do Rosário[2] and João Cajaiba[1,*]

[1] Universidade Federal do Rio de Janeiro (UFRJ), Instituto de Química, Pólo de Xistoquímica, Rua Hélio de Almeida 40, Cidade Universitária, Rio de Janeiro, 21941-614, Brazil.

[2] Centro de Pesquisas e Desenvolvimento Leopoldo Américo Miguez de Mello, PETROBRAS, Cidade Universitária, Rio de Janeiro 21040-000, Brazil.

* Correspondence: cajaiba@iq.ufrj.br; Tel.: +55-21-2562-5323

Academic Editor: name
Received: date; Accepted: date; Published: date

1. Introduction

In this supporting information, results regarding the regression models will be displayed. Even though the methods are common and basic in the statistics area, in order for a better understanding, a quick review regarding the statistical tests to evaluate any regression model will be given. We encourage the reader to consult other references for a deeper view of the methods discussed here.

1.1. Analysis of Variance (ANOVA)

Having been made a regression model, it is necessary to verify if there is a good fit between it and the experimental data. One of the most used method is the Analysis of Variance (ANOVA). In ANOVA, the variability of the experimental data is divided into two components:

• Sum of Squares of the Regression (SSR) - measures the amount of variability explained by the regression model;

• Sum of Squares of the Error (SSE) - measures the residual variation that is not explained by the independent variables;

We can then write the total variability of the experimental data as the Total Sum of Squares (SST) as in Equation (S1):

$$SST = \sum y_i^2 - \frac{(\sum y_i)^2}{N} = SSR + SSE \quad \text{(S1)}$$

where y_i is the i-th value of the dependent variable dataset and N is the number of experiments performed and used in the regression. The SST is correlated to how the experimental values (of the dependent variable) vary in respect to their mean value. Regarding the degree of freedom for each of the sums described, one can initially assume that the entire modelling has (N - 1) degrees of freedom. Hence, the SST has (N - 1) degrees of freedom. By doing the regression, p degrees are used for the estimation of the unknown coefficients (b_1, b_2, ..., b_p), where the linear coefficient b_0 is not considered a regressor since it is a function of other estimates. In this case, p is the total number of independent variables used in the mathematical model. Hence, the SSR has p degrees

of freedom. Since SST = SSR + SSE, then it can be found that the SSE has (*N - p - 1*) degrees of freedom. The SSE deals with the variability not explained by the regression, and can be defined as:

$$SSE = \sum(y_i - \hat{y}_i)^2 \quad \text{(S2)}$$

where $\hat{y}_i$ is the value predicted for the dependent variable by the model for the i-th sample.

The SSR relates the modelled data to the mean value of the dependent variable, and can be defined as:

$$SSR = \sum y_i^2 - \frac{(\sum \bar{y}_i)^2}{N} \quad \text{(S3)}$$

The sum of squares can be viewed as variances for comparison amongst themselves, by dividing them by their degrees of freedom. This leads to the mean squares (MS) values:

$$MSE = \frac{SSE}{N-p-1} \quad \text{(S4)}$$

$$MSR = \frac{SSR}{p} \quad \text{(S5)}$$

In addition, when one has a data set with replicates, in which variance can be estimated, one can partition the SSE into two components: one related to the pure error of the system and another associated with the lack of fit of the proposed mathematical model to the experimental points. The sum of squares related to the pure error (SSPE) can be written according to Equation (S6):

$$SSPE = \sum_{j=1}^{c}\sum_{i=1}^{n_j}(y_{ij} - \bar{y}_j)^2 \quad \text{(S6)}$$

In the equation, n_j is the number of replicates of the same point and c is the number of points in the set containing replicates. The value of SSPE is related to the experimental variance itself measured over the points in replicates, and no information of the regression is used for its calculation. In the case of design of experiments with replicates at the central point, we have N total experiments performed, where (n_j - 1) are repetitions of the same point. Hence, there are only *m* different experiments. In this case, the SSPE has a degree of freedom equal to (*N - m*). Since the SSE is partitioned into the sum of squares of the pure error and of the lack of fit (SSLF), the latter can be found from Equations (S2) and (S6):

$$SSLF = SSE - SSPE \quad \text{(S7)}$$

In this case, the degrees of freedom for the SSLF can be found as (*m - p - 1*). Table S1 summarizes the ANOVA calculations.

Table S1. Summary of the variation source for the ANOVA, the notations, degrees of freedom and significance tests

Variation Source	Sum of Squares	Degrees of Freedom	Mean Squares	F_0
Regression	*SSR*	*p*	*MSR*	*MSR/MSE*
Error	*SSE*	*N - p - 1*	*MSE*	
(Lack of Fit)	*SSLF*	*m - p - 1*	*MSLF*	*MSLF/MSPE*
(Pure Error)	*SSPE*	*N - m*	*MSPE*	
Total	*SST*	*N - 1*		

1.2. F-Test to Evaluate the Significance of the Regression

The ANOVA F-test for the regression serves to check if the proposed model, which uses information from the predictor variables, is substantially better than a simple predictor: the mean value of the answers, $\bar{y}$, which does not depend on any of these vari. For this, a hypothesis test is used, where:

$$H_0: b_1 = b_2 = \cdots = b_p = 0$$

$$H_a: \text{At least one of the coefficients } b_1, b_2, \cdots, b_p \text{ is not zero}$$

The F-value can be found by the ratio between the mean squares of the regression and of the error, which has an F-distribution:

$$F = \frac{MSR}{MSE} \tag{S8}$$

The F-value can be compared to a tabled value, Fcrit, with degrees of freedom relative to that of the regression and that of the error, in that order. If F > Fcrit, the null hypothesis is not valid. Therefore, there is a correlation between the response and some of the coefficients, so that one can declare that the regression is significant.

1.3. F-Test to Assess Lack of Fit

The F-test to verify the lack of fit must be performed in order to evaluate if the mathematical model proposed to respond to the experimental variability is adequate. The initial assumption is that the response could be described using the model that follows:

$$\boldsymbol{y} = \boldsymbol{bX} + \boldsymbol{\varepsilon} \tag{S9}$$

where $\boldsymbol{X}$ is the design matrix, $\boldsymbol{b}$ is the vector containing the coefficients and $\boldsymbol{\varepsilon}$ is the error vector.

However, the real model describing the experimental variability is described by:

$$\boldsymbol{y} = \boldsymbol{bX} + \boldsymbol{b_2X_2} + \boldsymbol{\varepsilon} \tag{S10}$$

Thus, the estimate of the coefficients of the proposed model leads to inflated and biased values when the model does not fit well and $\boldsymbol{b_2} \neq 0$. The hypothesis test for this evaluation is described by:

$H_0: \boldsymbol{b_2} = 0$

$H_a: \boldsymbol{b_2} \neq 0$

The F-value can be found by the ratio between the mean squares of the lack of fit and of the pure error, which has an F-distribution:

$$F = \frac{MSLF}{MSPE} \tag{S11}$$

The F-value can be compared to a tabled value, Fcrit, with degrees of freedom relative to the lack of fit and the pure error, in that order. If F < Fcrit, the null hypothesis is valid. Therefore, it can be said that the estimated coefficients are not biased and that there is a good fit between the mathematical model and the response.

1.4. Coefficient of Determination

The coefficient of determination, also well known as R^2, is a statistical measure of the strength of a regression model according to the total variability explained by it. It is defined as the ratio of the sum of squares of the regression in relation to the total sum of squares:

$$R^2 = \frac{SSR}{SST} \tag{S12}$$

Since the R^2 value always increases as a new variable is added to a model, this value can be inflated artificially with the inclusion of more and more predictor variables, and then pass a false image on the actual fit of the model with such variables. For this, it can defined an adjusted coefficient of determination by dividing the sum of squares by their corresponding degree of freedom:

$$R^2(adj.) = \frac{MSR}{SST/(N-1)} \quad \text{(S13)}$$

2. Methodology

The applied design of experiments used was a 2^{5-1} rotational central composite design, which design can be divided into three experimental portions: (1) factorial points, (2) central points, and (3) star points. The factorial portion was indeed a fractional factorial design with resolution V, meaning that all two factor interactions were aliased with three factor interactions. The central points were replicated six times. The star portion were made in order that the model would be rotational. Hence, α was chosen for this type of 2^{5-1} design. Table S2 shows the coded design matrix and Table S3 shows the information of the factors, and their intervals.

Table S2. Design matrix of the coded factors for the 2^{5-1} rotational central composite design

	Experiment #	Pressure (X_1)	Temperature (X_2)	MEG Conc. (X_3)	Bicarbonate Conc. (X_4)	Calcium Conc. (X_5)
Factorial Points	1	-1	-1	-1	-1	1
	2	1	-1	-1	-1	-1
	3	-1	1	-1	-1	-1
	4	1	1	-1	-1	1
	5	-1	-1	1	-1	-1
	6	1	-1	1	-1	1
	7	-1	1	1	-1	1
	8	1	1	1	-1	-1
	9	-1	-1	-1	1	-1
	10	1	-1	-1	1	1
	11	-1	1	-1	1	1
	12	1	1	-1	1	-1
	13	-1	-1	1	1	1
	14	1	-1	1	1	-1
	15	-1	1	1	1	-1
	16	1	1	1	1	1
Central Points	17	0	0	0	0	0
	18	0	0	0	0	0
	19	0	0	0	0	0
	20	0	0	0	0	0
	21	0	0	0	0	0
	22	0	0	0	0	0
Star Points	23	-2.378	0	0	0	0
	24	2.378	0	0	0	0
	25	0	-2.378	0	0	0
	26	0	2.378	0	0	0
	27	0	0	-2.378	0	0
	28	0	0	2.378	0	0
	29	0	0	0	-2.378	0
	30	0	0	0	2.378	0
	31	0	0	0	0	-2.378
	32	0	0	0	0	2.378

Table S3. Summary of the factors studied in the design of experiments. with their different levels studied for the modelling

	levels				
	-2.378	-1	0	1	2.378
X1: Pressure (psi)	0	714	1233	1751	2466
X2: Temperature (°C)	40	60	75	90	110
X3: MEG Conc. (%)	0	23	40	57	80
X4: Bicarbonate Conc. (ppm)	1000	2449	3500	4551	6000
X5: Calcium Conc. (ppm)	1000	2449	3500	4551	6000

3. Results

3.1. Response Matrix Used for the Modelling

The response matrix is presented at Table S4, where the scaling time, in seconds, is shown for the 32 central composite experiments. Each experiment shows 25 datapoints, corresponding to the 25 different models being constructed. It is worth remembering that the models are referenced to the advancement of the scaling process, and are related to how the differential pressure signal increases as deposition occurs in the tube line. Hence, Model 1 is the scaling time so that the baseline increases 1 psi; Model 2 is the scaling time so that the baseline increases 2 psi; and so on, until Model 25, which is the scaling time so that the baseline increases 25 psi.

3.2. Modelling of the Data Using the Scaling Time Directly

The first model tested was using the scaling time (t_{sc}) directly, using Equation 5 of the manuscript. However, variables (b_1, b_{12}, b_{11}, and b_{55}) did not show significance and were eliminated from the model. The final model was then:

$$\begin{aligned} t_{sc} = b_0 + (b_2 \times T) + \ (b_3 \times MEG) + (b_4 \times C_{HCO_3^-}) + (b_5 \times C_{Ca^{2+}}) + \ (b_{13} \times P \times MEG) \\ + (b_{14} \times P \times C_{HCO_3^-}) + (b_{15} \times P \times C_{Ca^{2+}}) + (b_{23} \times T \times MEG) + (b_{24} \times T \times C_{HCO_3^-}) \\ + (b_{25} \times P \times C_{Ca^{2+}}) + (b_{34} \times MEG \times C_{HCO_3^-}) + (b_{35} \times MEG \times C_{Ca^{2+}}) \\ + (b_{45} \times C_{HCO_3^-} \times C_{Ca^{2+}}) + (b_{22} \times T \times T) + (b_{33} \times MEG \times MEG) \\ + \left(b_{44} \times C_{HCO_3^-} \times C_{HCO_3^-}\right) \end{aligned}$$

The ANOVA results for the modelling using this equation are presented in Table S5, the calculated coefficients are presented in Table S6 and the p-value for these coefficients are shown in Table S7.

Table S4. Response matrix showing the measured scaling time (in seconds) for the different experiments in the central composite design for the different models constructed

	Model Number																								
**Experiment #**	**1**	**2**	**3**	**4**	**5**	**6**	**7**	**8**	**9**	**10**	**11**	**12**	**13**	**14**	**15**	**16**	**17**	**18**	**19**	**20**	**21**	**22**	**23**	**24**	**25**
1	1387	1444	1470	1487	1497	1504	1510	1514	1518	1520	1522	1524	1526	1528	1529	1531	1532	1533	1534	1535	1536	1537	1538	1538	1539
2	1728	1862	1917	1948	1968	1983	1996	2004	2011	2016	2021	2025	2029	2032	2035	2038	2041	2043	2045	2047	2049	2051	2052	2054	2055
3	431	434	436	437	438	439	440	440	441	441	442	442	442	443	443	443	444	444	444	444	444	445	445	445	445
4	379	423	432	434	436	437	438	439	439	440	440	440	441	441	441	442	442	442	443	443	443	443	443	444	444
5	2506	2690	2752	2782	2799	2811	2820	2828	2834	2839	2843	2847	2850	2853	2856	2859	2861	2864	2866	2868	2869	2871	2872	2874	2876
6	1937	1994	2038	2066	2080	2091	2099	2107	2116	2124	2131	2137	2142	2147	2151	2154	2157	2160	2162	2164	2168	2170	2173	2174	2176
7	1265	1374	1423	1452	1475	1493	1505	1519	1529	1536	1545	1553	1559	1564	1570	1574	1576	1589	1592	1594	1599	1601	1603	1605	1607
8	1270	1363	1412	1443	1458	1474	1485	1494	1501	1507	1512	1516	1520	1524	1526	1529	1531	1533	1535	1537	1538	1540	1541	1542	1543
9	865	900	917	931	942	950	955	960	964	967	970	972	973	975	976	978	979	980	981	982	983	984	985	985	986
10	957	984	996	1003	1008	1012	1014	1017	1018	1020	1021	1022	1023	1024	1025	1026	1026	1027	1028	1028	1029	1029	1030	1030	1030
11	452	458	460	461	462	463	464	464	465	465	466	466	466	467	467	467	467	468	468	468	468	468	468	469	469
12	397	403	405	407	408	408	409	409	410	410	411	411	411	412	412	412	412	412	413	413	413	413	413	413	414
13	2826	3204	3326	3394	3418	3473	3488	3497	3502	3507	3510	3514	3518	3521	3523	3525	3526	3528	3529	3530	3531	3532	3533	3534	3535
14	2810	3245	3478	3623	3729	3811	3876	3924	3962	3998	4020	4033	4045	4055	4069	4116	4127	4136	4145	4153	4159	4165	4172	4188	4194
15	1084	1128	1167	1193	1216	1224	1237	1243	1262	1273	1284	1306	1308	1320	1335	1344	1349	1361	1369	1381	1378	1380	1381	1381	1382
16	1137	1218	1250	1281	1292	1305	1309	1312	1325	1328	1330	1340	1342	1343	1345	1346	1348	1349	1351	1353	1355	1357	1358	1360	1361
17	640	658	667	672	677	680	683	685	686	688	689	691	692	694	695	697	698	699	700	701	701	702	703	703	704
18	735	758	768	775	780	784	787	790	792	795	797	798	800	802	803	804	806	807	808	809	810	811	811	812	813
19	670	682	688	692	695	696	698	699	700	701	702	703	705	706	706	707	708	709	709	710	710	711	711	711	712
20	741	766	775	779	782	785	786	788	790	791	792	793	794	796	797	798	799	799	800	800	800	801	801	801	802
21	767	781	785	788	791	793	794	795	798	799	800	802	802	803	804	805	805	806	807	807	808	808	809	809	809
22	675	688	695	698	699	701	702	703	704	704	705	706	706	707	708	708	709	709	709	710	710	711	711	711	712
23	730	791	820	836	847	857	865	871	876	880	884	887	891	893	896	898	901	903	904	906	908	910	911	912	914
24	738	759	769	774	777	780	782	784	786	788	789	790	792	793	793	794	795	795	796	797	797	798	798	799	799
25	5922	6197	6276	6322	6353	6377	6399	6417	6432	6445	6456	6467	6477	6485	6494	6501	6508	6514	6519	6525	6530	6533	6538	6542	6545
26	642	651	653	654	655	656	656	657	658	658	658	662	665	665	666	666	666	666	667	667	667	667	667	668	668
27	613	631	638	642	644	647	648	650	652	653	654	655	656	657	658	658	659	660	660	661	661	662	662	663	663
28	4776	5348	5566	5753	5860	5908	5964	6000	6023	6052	6073	6103	6123	6149	6152	6162	6171	6182	6193	6204	6207	6208	6211	6213	6222
29	1197	1268	1299	1318	1331	1341	1350	1357	1363	1368	1373	1377	1381	1385	1388	1392	1395	1398	1401	1404	1407	1409	1412	1414	1417
30	607	616	619	621	623	624	625	626	627	627	629	630	631	632	632	632	633	633	634	634	634	634	635	635	635
31	940	986	1006	1018	1027	1033	1038	1042	1045	1049	1051	1054	1056	1059	1060	1062	1064	1065	1067	1068	1070	1071	1072	1073	1074
32	765	796	809	815	819	822	825	828	833	851	853	855	856	857	858	858	859	861	861	862	863	863	864	864	865

Table S5. ANOVA results for the equation using the scaling time as response

Model Number	R^2	R^2 adj.	F-value Regression[(a)]	F-value Lack-of-Fit[(b)]
1	0.927	0.848	11.9	224.54
2	0.934	0.863	13.3	181.60
3	0.938	0.872	14.2	157.79
4	0.939	0.874	14.5	148.04
5	0.940	0.875	14.6	141.92
6	0.941	0.879	15.0	131.65
7	0.942	0.879	15.1	127.60
8	0.942	0.880	15.2	123.30
9	0.942	0.880	15.3	118.92
10	0.942	0.880	15.3	116.77
11	0.942	0.880	15.3	114.61
12	0.942	0.880	15.2	114.33
13	0.942	0.880	15.2	112.32
14	0.942	0.880	15.1	112.75
15	0.942	0.880	15.2	110.76
16	0.942	0.881	15.3	109.69
17	0.942	0.881	15.3	108.43
18	0.942	0.881	15.3	107.77
19	0.942	0.881	15.3	107.29
20	0.942	0.880	15.3	107.28
21	0.942	0.880	15.3	105.45
22	0.942	0.881	15.3	104.76
23	0.942	0.881	15.3	104.43
24	0.942	0.881	15.3	103.26
25	0.942	0.881	15.3	103.06

(a) F-crit = 2.65 for a significance level of 0.95

(b) F-crit = 4.95 for a significance level of 0.95

Table S6. Coefficients calculated in the regressions for the different models using the scaling time as response

	**Coefficients**																
**Model Number**	b_0	b_2	b_3	b_4	b_5	b_{13}	b_{14}	b_{15}	b_{23}	b_{24}	b_{25}	b_{34}	b_{35}	b_{45}	b_{22}	b_{33}	b_{44}
1	659.9	-774.7	664.2	-65.1	-42.7	-53.3	21.8	-177.4	-127.8	-10.9	53.3	133.3	-16.2	73.9	437.0	333.1	16.1
2	689.6	-831.6	751.6	-58.4	-50.5	-63.3	28.8	-223.9	-161.1	-45.6	76.0	174.5	-21.6	81.4	457.9	381.1	19.0
3	705.4	-852.5	788.4	-54.9	-57.0	-59.8	33.8	-243.9	-173.4	-60.1	86.2	192.1	-28.4	76.2	463.7	399.6	20.6
4	713.0	-864.3	815.8	-51.8	-61.1	-55.3	37.6	-255.5	-179.1	-68.3	92.6	203.3	-31.9	72.3	466.8	415.5	21.5
5	718.3	-871.7	831.6	-49.8	-65.3	-51.9	41.6	-262.8	-182.1	-73.9	98.8	210.1	-36.5	66.3	468.9	424.4	22.1
6	724.3	-878.7	842.1	-47.3	-66.8	-50.3	43.0	-271.3	-186.8	-81.3	101.8	217.1	-37.1	65.1	470.9	428.6	22.7
7	727.6	-883.5	850.8	-46.3	-69.5	-48.1	45.1	-276.3	-188.9	-84.8	105.1	221.4	-40.2	61.7	472.4	433.2	23.2
8	730.5	-887.2	856.8	-46.0	-71.1	-46.3	47.2	-279.9	-190.3	-88.1	108.1	223.4	-42.2	58.8	473.6	436.2	23.5
9	733.8	-889.2	861.9	-45.1	-72.4	-44.6	48.6	-281.2	-190.1	-88.4	110.1	226.4	-43.8	56.1	474.8	438.1	23.8
10	737.4	-891.7	866.8	-44.6	-72.6	-43.2	49.6	-282.9	-191.1	-90.1	111.7	228.4	-45.8	53.4	475.3	440.1	23.6
11	739.2	-893.3	870.3	-44.4	-73.6	-42.6	50.0	-284.1	-191.1	-90.8	112.6	229.4	-46.8	51.4	476.0	441.8	24.0
12	740.8	-893.6	875.1	-43.8	-74.3	-43.0	49.8	-283.5	-189.5	-89.8	112.8	231.3	-47.5	49.8	477.0	444.2	24.1
13	741.9	-894.8	877.8	-44.2	-74.6	-42.7	50.1	-284.6	-189.7	-90.4	113.4	231.7	-47.7	49.1	477.8	445.7	24.2
14	743.1	-895.7	881.2	-44.1	-75.4	-42.8	49.7	-284.8	-189.3	-90.3	113.2	232.3	-48.4	47.8	478.2	447.8	24.3
15	744.6	-896.5	882.9	-43.8	-76.2	-43.2	49.7	-284.9	-188.8	-90.2	113.4	233.6	-49.6	46.1	479.0	448.0	24.5
16	746.7	-898.8	886.1	-42.6	-78.3	-41.0	51.9	-287.5	-190.8	-92.6	116.0	236.6	-52.8	42.6	479.5	448.9	24.8
17	747.9	-899.8	887.6	-42.6	-79.0	-40.5	52.1	-287.9	-190.9	-92.8	116.5	237.5	-53.3	41.9	479.9	449.5	24.9
18	749.1	-900.0	889.9	-42.7	-79.2	-41.4	52.4	-288.4	-189.9	-93.1	117.1	237.7	-53.7	40.2	480.3	450.4	25.1
19	749.9	-900.4	891.6	-42.5	-79.9	-41.6	52.6	-288.6	-189.7	-93.1	117.3	238.4	-54.4	39.2	480.7	451.3	25.3
20	750.8	-900.9	893.5	-42.3	-80.6	-41.8	52.3	-288.4	-189.1	-92.6	117.3	239.5	-55.4	38.1	481.1	452.2	25.4
21	751.5	-901.8	894.2	-42.8	-80.4	-41.3	52.9	-289.0	-189.3	-93.1	118.0	239.3	-54.9	37.8	481.4	452.3	25.5
22	752.6	-902.3	894.7	-42.9	-80.8	-40.9	53.1	-289.3	-189.3	-93.3	118.4	239.6	-55.0	37.5	481.5	452.4	25.6
23	753,1	-903,1	895,4	-43,0	-80,9	-40,4	53,4	-289,6	-189,4	-93,6	118,7	239,8	-55,2	36,9	481,9	452,5	25,8
24	753,7	-903,9	896,3	-42,8	-81,5	-39,6	54,4	-290,6	-190,4	-94,4	120,1	240,8	-56,0	36,3	482,3	452,8	26,0
25	754,5	-904,5	897,5	-43,0	-81,7	-39,4	54,8	-291,0	-190,6	-94,5	120,5	240,9	-56,1	35,8	482,4	453,4	26,1

Table S7. P-value calculated for the coefficients in the regressions for the different models using the scaling time as response

	**p-value[(a)]**																
**Model Number**	$\mathbf{b_0}$	$\mathbf{b_2}$	$\mathbf{b_3}$	$\mathbf{b_4}$	$\mathbf{b_5}$	$\mathbf{b_{13}}$	$\mathbf{b_{14}}$	$\mathbf{b_{15}}$	$\mathbf{b_{23}}$	$\mathbf{b_{24}}$	$\mathbf{b_{25}}$	$\mathbf{b_{34}}$	$\mathbf{b_{35}}$	$\mathbf{b_{45}}$	$\mathbf{b_{22}}$	$\mathbf{b_{33}}$	$\mathbf{b_{44}}$
1	0.000	0.000	0.000	0.000	0.000	0.000	0.069	0.000	0.000	0.340	0.000	0.000	0.166	0.000	0.000	0.000	0.018
2	0.000	0.000	0.000	0.000	0.000	0.000	0.041	0.000	0.000	0.003	0.000	0.000	0.112	0.000	0.000	0.000	0.016
3	0.000	0.000	0.000	0.000	0.000	0.001	0.027	0.000	0.000	0.001	0.000	0.000	0.057	0.000	0.000	0.000	0.015
4	0.000	0.000	0.000	0.000	0.000	0.002	0.020	0.000	0.000	0.000	0.000	0.000	0.043	0.000	0.000	0.000	0.015
5	0.000	0.000	0.000	0.001	0.000	0.003	0.014	0.000	0.000	0.000	0.000	0.000	0.027	0.001	0.000	0.000	0.015
6	0.000	0.000	0.000	0.001	0.000	0.005	0.014	0.000	0.000	0.000	0.000	0.000	0.029	0.001	0.000	0.000	0.016
7	0.000	0.000	0.000	0.002	0.000	0.008	0.012	0.000	0.000	0.000	0.000	0.000	0.022	0.001	0.000	0.000	0.016
8	0.000	0.000	0.000	0.002	0.000	0.011	0.010	0.000	0.000	0.000	0.000	0.000	0.019	0.002	0.000	0.000	0.016
9	0.000	0.000	0.000	0.003	0.000	0.016	0.010	0.000	0.000	0.000	0.000	0.000	0.017	0.004	0.000	0.000	0.017
10	0.000	0.000	0.000	0.003	0.000	0.020	0.009	0.000	0.000	0.000	0.000	0.000	0.014	0.006	0.000	0.000	0.019
11	0.000	0.000	0.000	0.004	0.000	0.022	0.009	0.000	0.000	0.000	0.000	0.000	0.014	0.008	0.000	0.000	0.018
12	0.000	0.000	0.000	0.004	0.000	0.022	0.010	0.000	0.000	0.000	0.000	0.000	0.013	0.010	0.000	0.000	0.019
13	0.000	0.000	0.000	0.004	0.000	0.024	0.010	0.000	0.000	0.000	0.000	0.000	0.014	0.012	0.000	0.000	0.019
14	0.000	0.000	0.000	0.004	0.000	0.024	0.011	0.000	0.000	0.000	0.000	0.000	0.013	0.014	0.000	0.000	0.019
15	0.000	0.000	0.000	0.005	0.000	0.024	0.011	0.000	0.000	0.000	0.000	0.000	0.012	0.017	0.000	0.000	0.019
16	0.000	0.000	0.000	0.006	0.000	0.031	0.009	0.000	0.000	0.000	0.000	0.000	0.008	0.026	0.000	0.000	0.018
17	0.000	0.000	0.000	0.006	0.000	0.034	0.009	0.000	0.000	0.000	0.000	0.000	0.008	0.029	0.000	0.000	0.019
18	0.000	0.000	0.000	0.006	0.000	0.032	0.009	0.000	0.000	0.000	0.000	0.000	0.008	0.036	0.000	0.000	0.018
19	0.000	0.000	0.000	0.007	0.000	0.032	0.009	0.000	0.000	0.000	0.000	0.000	0.007	0.041	0.000	0.000	0.018
20	0.000	0.000	0.000	0.007	0.000	0.031	0.010	0.000	0.000	0.000	0.000	0.000	0.007	0.047	0.000	0.000	0.018
21	0.000	0.000	0.000	0.007	0.000	0.035	0.010	0.000	0.000	0.000	0.000	0.000	0.008	0.050	0.000	0.000	0.018
22	0.000	0.000	0.000	0.007	0.000	0.036	0.009	0.000	0.000	0.000	0.000	0.000	0.008	0.052	0.000	0.000	0.018
23	0.000	0.000	0.000	0.007	0.000	0.039	0.009	0.000	0.000	0.000	0.000	0.000	0.008	0.056	0.000	0.000	0.018
24	0.000	0.000	0.000	0.007	0.000	0.043	0.009	0.000	0.000	0.000	0.000	0.000	0.007	0.061	0.000	0.000	0.018
25	0.000	0.000	0.000	0.007	0.000	0.045	0.008	0.000	0.000	0.000	0.000	0.000	0.007	0.065	0.000	0.000	0.017

(a) p-values were calculated for a significance level of 0.95

Results showed good coefficient of determination and the models were approved as significant for the regression F-test (F > F-crit). However, the models were not approved by the lack of fit F-test (F > F-crit). This means that the mathematical equation proposed was not good to represent the experimental variability. Evaluating the residuals graph for model 1 (Figure S1), it can be seen a trend, which indicates that the data is non-linear.

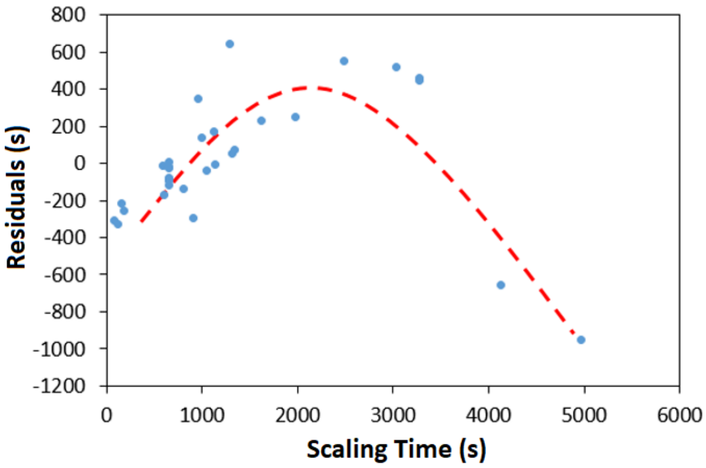

Figure S1. Graph of residuals versus predicted value for model 1

If data if non-linear, a way to overcome is by trying to make it linear. A transformation was then made by using the logarithm version of the response matrix (Table S4).

3.2. Modelling of the Data Using the Natural Logarithm of the Scaling Time

The second model tested was using the natural logarithm of the scaling time ($\ln(t_{sc})$), as presented in Equation (5) of the manuscript. However, variables (b_1, b_{12}, b_{13}, b_{14}, b_{24}, and b_{35}) did not show significance and were eliminated from the model. The final model was:

$$\ln(t_{sc}) = b_0 + (b_2 \times T) + (b_3 \times MEG) + (b_4 \times C_{HCO_3^-}) + (b_5 \times C_{Ca^{2+}}) + (b_{15} \times P \times C_{Ca^{2+}}) + (b_{23} \times T \times MEG) + (b_{25} \times P \times C_{Ca^{2+}}) + (b_{34} \times MEG \times C_{HCO_3^-}) + (b_{45} \times C_{HCO_3^-} \times C_{Ca^{2+}}) + (b_{11} \times P \times P) + (b_{22} \times T \times T) + (b_{33} \times MEG \times MEG) + \left(b_{44} \times C_{HCO_3^-} \times C_{HCO_3^-}\right) + (b_{55} \times C_{Ca^{2+}} \times C_{Ca^{2+}})$$

The ANOVA results for the modelling using this equation are presented in Table S8, the calculated coefficients are presented in Table S9 and the p-value for these coefficients are shown in Table S10.

Table S8. ANOVA results for the equation using the natural logarithm of the scaling time as response

Model Number	R^2	R^2 adj.	F-value Regression[(a)]	F-value Lack-of-Fit[(b)]
1	0.991	0.984	139.6	1.65
2	0.991	0.983	131.7	1.82
3	0.990	0.982	123.0	2.11
4	0.990	0.981	117.4	2.29
5	0.989	0.981	113.3	2.43
6	0.989	0.980	109.5	2.52
7	0.989	0.980	107.4	2.64
8	0.989	0.979	106.2	2.67
9	0.988	0.979	103.1	2.69
10	0.988	0.979	102.6	2.69

Table S8. *Cont.*

11	0.988	0.978	101.4	2.72
12	0.988	0.978	99.6	2.80
13	0.988	0.978	100.0	2.80
14	0.988	0.978	98.9	2.84
15	0.988	0.977	96.8	2.90
16	0.987	0.977	94.8	2.99
17	0.987	0.977	94.7	3.00
18	0.987	0.976	92.9	3.08
19	0.987	0.976	92.1	3.07
20	0.987	0.976	91.0	3.17
21	0.987	0.976	90.8	3.13
22	0.987	0.976	90.7	3.17
23	0.987	0.976	90.6	3.18
24	0.987	0.976	90.0	3.18
25	0.987	0.976	89.8	3.22

(a) F-crit = 2.33 for a significance level of 0.95

(b) F-crit = 4.68 for a significance level of 0.95

Table S9. Coefficients calculated in the regressions for the different models using the natural logarithm of the scaling time as response

	Coefficients														
**Model Number**	b_0	b_2	b_3	b_4	b_5	b_{15}	b_{23}	b_{25}	b_{34}	b_{45}	b_{11}	b_{22}	b_{33}	b_{44}	b_{55}
1	6.560	-0.457	0.442	-0.086	-0.030	-0.120	0.078	0.026	0.075	0.056	0.010	0.183	0.160	0.037	0.036
2	6.587	-0.465	0.460	-0.090	-0.029	-0.130	0.074	0.042	0.091	0.053	0.017	0.185	0.170	0.040	0.041
3	6.598	-0.468	0.469	-0.091	-0.031	-0.135	0.075	0.045	0.096	0.050	0.020	0.186	0.173	0.042	0.043
4	6.605	-0.469	0.475	-0.091	-0.033	-0.137	0.077	0.047	0.099	0.048	0.022	0.186	0.176	0.043	0.044
5	6.609	-0.470	0.479	-0.091	-0.034	-0.139	0.079	0.049	0.101	0.046	0.023	0.186	0.177	0.043	0.044
6	6.613	-0.471	0.481	-0.091	-0.035	-0.141	0.080	0.050	0.102	0.045	0.024	0.186	0.178	0.043	0.045
7	6.616	-0.472	0.483	-0.091	-0.036	-0.142	0.080	0.051	0.103	0.044	0.024	0.186	0.178	0.044	0.045
8	6.618	-0.473	0.484	-0.091	-0.036	-0.143	0.081	0.052	0.103	0.043	0.025	0.186	0.179	0.044	0.045
9	6.620	-0.472	0.485	-0.091	-0.036	-0.142	0.083	0.052	0.105	0.042	0.025	0.186	0.179	0.044	0.046
10	6.622	-0.472	0.487	-0.091	-0.035	-0.142	0.083	0.052	0.105	0.040	0.025	0.186	0.179	0.044	0.048
11	6.624	-0.472	0.487	-0.091	-0.036	-0.142	0.084	0.052	0.105	0.039	0.026	0.185	0.180	0.045	0.048
12	6.625	-0.471	0.489	-0.090	-0.036	-0.141	0.086	0.052	0.106	0.038	0.026	0.186	0.180	0.045	0.048
13	6.627	-0.471	0.489	-0.090	-0.036	-0.141	0.086	0.052	0.106	0.038	0.026	0.186	0.180	0.045	0.048
14	6.629	-0.471	0.490	-0.090	-0.037	-0.141	0.086	0.052	0.106	0.037	0.026	0.186	0.180	0.045	0.048
15	6.630	-0.471	0.491	-0.090	-0.037	-0.141	0.087	0.051	0.107	0.036	0.026	0.186	0.180	0.045	0.048
16	6.631	-0.471	0.491	-0.090	-0.038	-0.141	0.087	0.052	0.108	0.035	0.026	0.186	0.180	0.045	0.048
17	6.633	-0.471	0.492	-0.090	-0.038	-0.141	0.088	0.052	0.108	0.035	0.027	0.186	0.180	0.045	0.048
18	6.634	-0.471	0.492	-0.090	-0.038	-0.141	0.089	0.052	0.108	0.034	0.027	0.186	0.180	0.045	0.048
19	6.635	-0.470	0.493	-0.090	-0.038	-0.141	0.089	0.052	0.108	0.033	0.027	0.186	0.180	0.045	0.048
20	6.635	-0.470	0.493	-0.090	-0.039	-0.141	0.090	0.052	0.109	0.033	0.027	0.186	0.180	0.045	0.048
21	6.636	-0.470	0.493	-0.090	-0.039	-0.141	0.090	0.052	0.109	0.033	0.027	0.186	0.180	0.046	0.049
22	6.637	-0.470	0.493	-0.090	-0.039	-0.141	0.090	0.052	0.109	0.033	0.027	0.186	0.180	0.045	0.048
23	6.637	-0.470	0.494	-0.090	-0.039	-0.141	0.090	0.052	0.109	0.032	0.027	0.186	0.180	0.046	0.049
24	6.638	-0.470	0.494	-0.091	-0.039	-0.141	0.090	0.053	0.109	0.032	0.027	0.186	0.180	0.046	0.049
25	6.639	-0.470	0.494	-0.091	-0.039	-0.141	0.090	0.053	0.109	0.032	0.027	0.186	0.180	0.046	0.049

Table S10. P-value calculated for the coefficients in the regressions for the different models using the natural logarithm of the scaling time as response

	p-value[a]													
**Model Number**	**b_0**	**b_2**	**b_3**	**b_4**	**b_5**	**b_{15}**	**b_{23}**	**b_{25}**	**b_{34}**	**b_{45}**	**b_{11}**	**b_{22}**	**b_{33}**	**b_{44}**	**b_{55}**
1	0.000	0.000	0.000	0.000	0.041	0.000	0.000	0.135	0.001	0.006	0.225	0.000	0.000	0.002	0.002
2	0.000	0.000	0.000	0.000	0.048	0.000	0.001	0.035	0.000	0.010	0.101	0.000	0.000	0.001	0.001
3	0.000	0.000	0.000	0.000	0.035	0.000	0.001	0.022	0.000	0.013	0.058	0.000	0.000	0.001	0.001
4	0.000	0.000	0.000	0.000	0.028	0.000	0.000	0.018	0.000	0.015	0.043	0.000	0.000	0.001	0.000
5	0.000	0.000	0.000	0.000	0.022	0.000	0.000	0.014	0.000	0.021	0.037	0.000	0.000	0.001	0.000
6	0.000	0.000	0.000	0.000	0.022	0.000	0.000	0.013	0.000	0.022	0.031	0.000	0.000	0.000	0.000
7	0.000	0.000	0.000	0.000	0.018	0.000	0.000	0.011	0.000	0.024	0.026	0.000	0.000	0.000	0.000
8	0.000	0.000	0.000	0.000	0.017	0.000	0.000	0.010	0.000	0.028	0.024	0.000	0.000	0.000	0.000
9	0.000	0.000	0.000	0.000	0.018	0.000	0.000	0.011	0.000	0.033	0.024	0.000	0.000	0.000	0.000
10	0.000	0.000	0.000	0.000	0.021	0.000	0.000	0.011	0.000	0.038	0.023	0.000	0.000	0.000	0.000
11	0.000	0.000	0.000	0.000	0.020	0.000	0.000	0.011	0.000	0.042	0.022	0.000	0.000	0.000	0.000
12	0.000	0.000	0.000	0.000	0.019	0.000	0.000	0.011	0.000	0.046	0.021	0.000	0.000	0.000	0.000
13	0.000	0.000	0.000	0.000	0.019	0.000	0.000	0.011	0.000	0.048	0.020	0.000	0.000	0.000	0.000
14	0.000	0.000	0.000	0.000	0.017	0.000	0.000	0.011	0.000	0.051	0.020	0.000	0.000	0.000	0.000
15	0.000	0.000	0.000	0.000	0.016	0.000	0.000	0.012	0.000	0.056	0.020	0.000	0.000	0.000	0.000
16	0.000	0.000	0.000	0.000	0.015	0.000	0.000	0.011	0.000	0.063	0.019	0.000	0.000	0.000	0.000
17	0.000	0.000	0.000	0.000	0.014	0.000	0.000	0.011	0.000	0.064	0.018	0.000	0.000	0.000	0.000
18	0.000	0.000	0.000	0.000	0.014	0.000	0.000	0.011	0.000	0.070	0.018	0.000	0.000	0.000	0.000
19	0.000	0.000	0.000	0.000	0.013	0.000	0.000	0.011	0.000	0.076	0.018	0.000	0.000	0.000	0.000
20	0.000	0.000	0.000	0.000	0.012	0.000	0.000	0.011	0.000	0.078	0.017	0.000	0.000	0.000	0.000
21	0.000	0.000	0.000	0.000	0.013	0.000	0.000	0.011	0.000	0.080	0.017	0.000	0.000	0.000	0.000
22	0.000	0.000	0.000	0.000	0.012	0.000	0.000	0.011	0.000	0.079	0.016	0.000	0.000	0.000	0.000
23	0.000	0.000	0.000	0.000	0.012	0.000	0.000	0.011	0.000	0.080	0.016	0.000	0.000	0.000	0.000
24	0.000	0.000	0.000	0.000	0.012	0.000	0.000	0.010	0.000	0.082	0.016	0.000	0.000	0.000	0.000
25	0.000	0.000	0.000	0.000	0.012	0.000	0.000	0.010	0.000	0.083	0.016	0.000	0.000	0.000	0.000

(a) p-values were calculated for a significance level of 0.95

Results showed good coefficient of determination, both close to each other and close to 1. The models were approved as significant for the regression F-test (F > F-crit). The models were also approved by the lack of fit F-test (F < F-crit). This means that the mathematical equation proposed was now good to represent the experimental variability and could be used for further evaluations.

2.3. Polymorphic Evaluation of the Systems Containing MEG

In order to evaluate if the MEG molecules are changing the polymorphic stabilization of calcium carbonate, a change in the usual dynamic scale loop methodology was made. For that, instead of the loop test, a high pressure filter was used, in order to sample the crystals that were being formed. A scheme of the equipment used for this evaluation is presented in Figure S2, and can be compared to the Figure 1 of the manuscript.

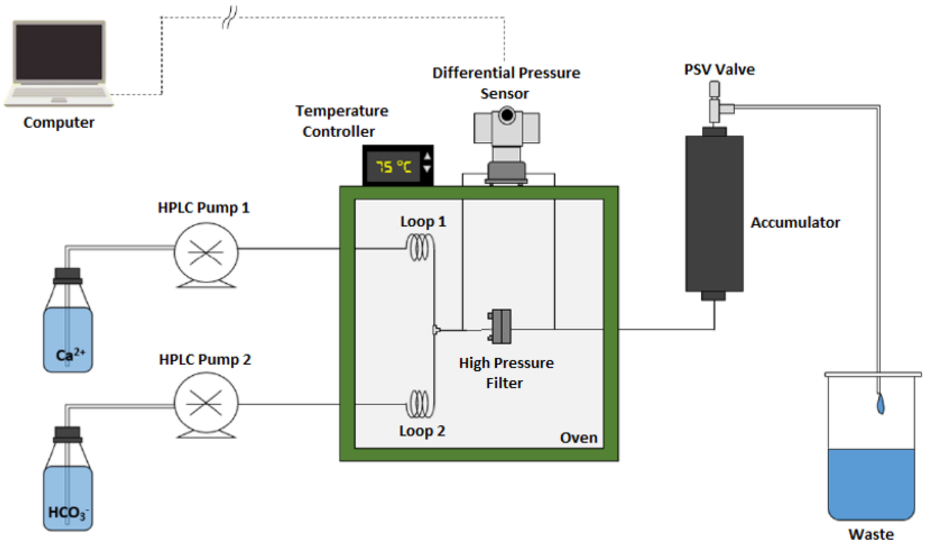

Figure S2. Scheme of the Modified Dynamic Scale Loop (DSL) system used in the solid sampling experiments

The experiments were performed with the same conditions as experiments #27 (0% MEG), #17-22 (40% MEG) and #28 (80% MEG), with the flow of each pump equal to 5 mL min^{-1}. For each experiment, the solid was retained on the filter for 10 minutes. After this time, the solutions were no longer pumped into the system and pressure was relieved. The solid was separated and dried in a vacuum oven for further analysis by scanning electron microscopy (SEM) imaging, using the Phenom ProX equipment (PhenomWorld, Eindhoven, Netherlands). Figures S3-S5 show the SEM images for the reactions performed with different MEG concentrations.

It could be seen that under the experimental conditions and without MEG, the major polymorph was aragonite (needle-shaped), with some crystals of calcite (cube shape). The addition of 40% MEG still led to the major precipitation of aragonite and calcite. This 40% concentration is the point at which MEG begins to affect the system in order to inhibit scale formation, so the comparison with the blank experiment did not lead to much difference. However, the experiment with the addition of 80% of MEG led to a more drastic change in the polymorphs formed, where aragonite was no longer the majority. Calcite was found in a greater amount, as well as vaterite, which came to be produced in the medium. Hence, it can be concluded that the inhibiting effect is also associated with a change in the stability of the different phases of calcium carbonate.

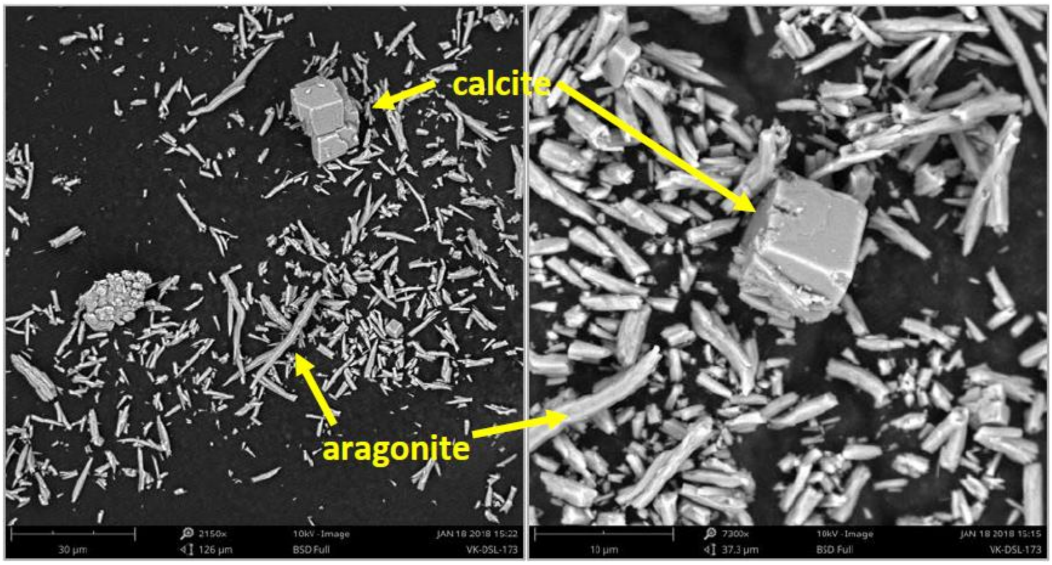

Figure S3. SEM images for the experiment containing 0% MEG with magnifications: (a) 2150x and (b) 7300x

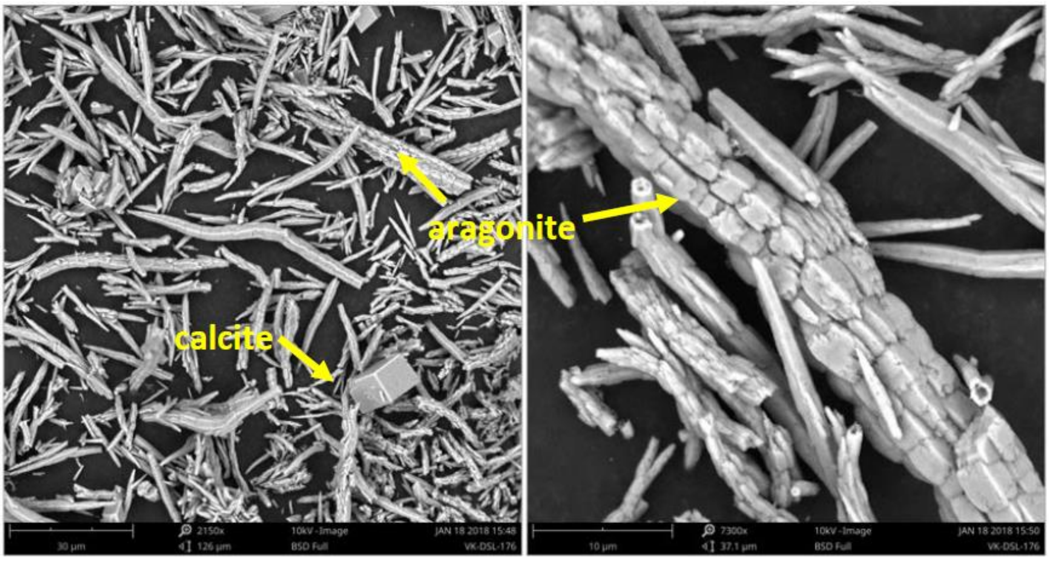

Figure S4. SEM images for the experiment containing 40% MEG with magnifications: (a) 2150x and (b) 7300x

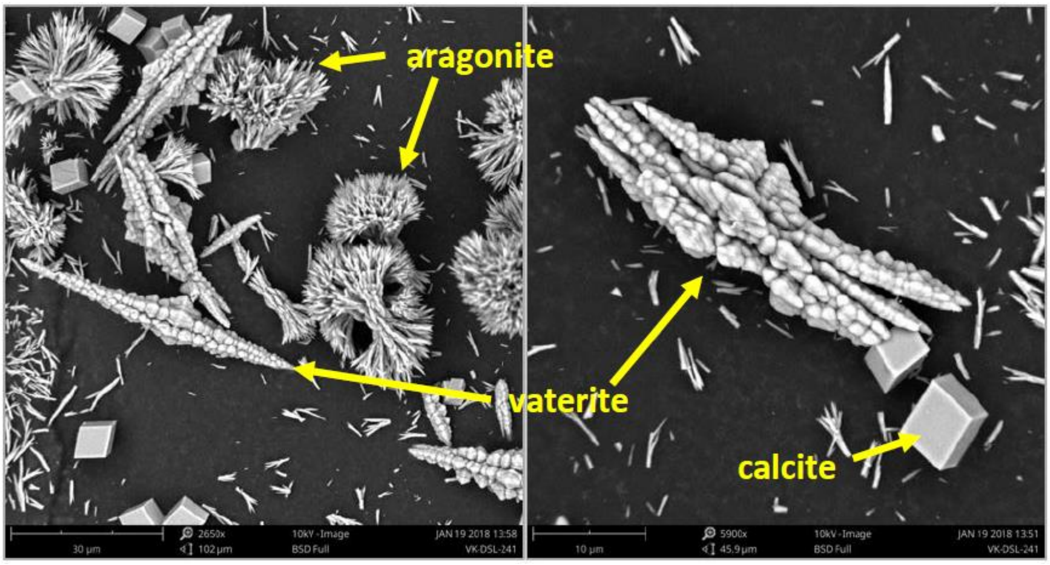

Figure S5. SEM images for the experiment containing 80% MEG with magnifications: (a) 2650x and (b) 5950x
